# A Rare Presentation of Chronic Vulval Sinus: A Case Report

**DOI:** 10.7759/cureus.48355

**Published:** 2023-11-06

**Authors:** Rabia Batool, Sasikala Selvamani, Chaudhri Shahbaz, Michael Slattery

**Affiliations:** 1 Obstetrics and Gynecology, Our Lady of Lourdes Hospital, Drogheda, IRL; 2 Surgery, Our Lady of Lourdes Hospital, Drogheda, IRL; 3 Radiology, Our Lady of Lourdes Hospital, Drogheda, IRL

**Keywords:** dysplasia, labiocrural fold, colposcopy, apocrine glands, sebum, hpv, pilonidal sinus, hidradenitis suppurativa, sinus tract, vulval sinus

## Abstract

We report an unusual case of the vulval sinus with persistent seroanguinous discharge in a 59-year-old postmenopausal woman. Examination revealed a 9-cm isolated sinus tract in the right labiocrural fold. This sinus tract was excised under anesthesia. Histological analysis showed skin with a deep dermal sinus tract showing edematous and heavily inflamed granulation tissue. There was no evidence of malignancy or dysplasia. Hidradenitis was ruled out on histology. This case report summarizes the importance of early diagnosis and prompt treatment involving complete excision of the vulval sinus to reduce long-term morbidity and consequences of this rare entity.

## Introduction

This article was previously presented as a poster at the BSCCP Annual Scientific Meeting 2023. A wound that fails to heal in six months or more is defined as a sinus [1]. A chronic discharging vulval sinus can be a distressing and embarrassing situation for women and warrants timely attention. This is a relatively rare condition affecting the female genital tract and is very rarely reported in developed countries. However, perineal sinus is much more common in developing countries [1]. It entails the formation of an abnormal opening in the vulva, causing persistent vulval discharge, and can be accompanied by pain, irritation, and inflammation. Hesitancy to seek timely help may lead to treatment delays resulting in abscess formation, scarring, and perineal disfigurement. Chronic abscesses that have been epithelialized eventually become fistula-in-ano [2]. Understanding the etiology, clinical manifestations, and therapeutic options for this condition is crucial for effective management and improving the quality of life. In this article, we delve into the intricacies of chronic discharging vulval sinuses, shedding light on the underlying factors and potential solutions.

## Case presentation

**Figure 1:**
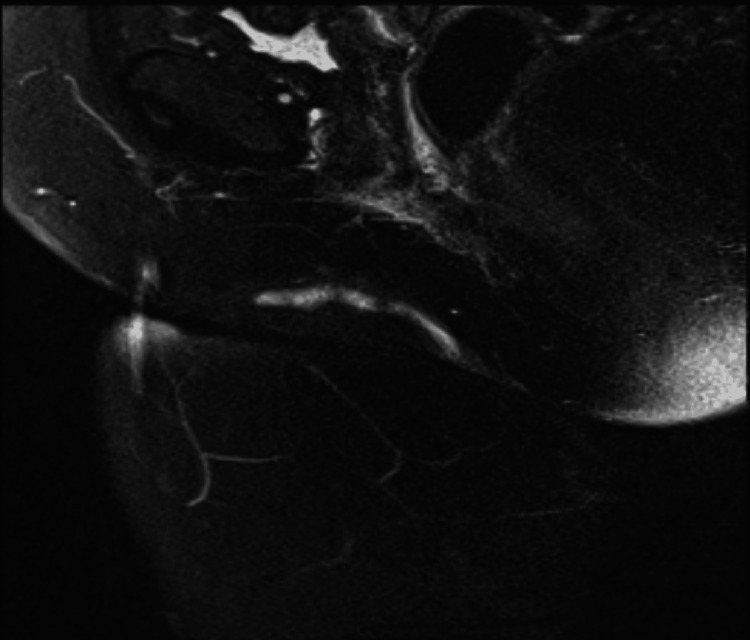
MRI Pelvis showing sinus tract

​​A 59-year-old woman, mother of three was referred to the gynecology outpatients for evaluation of persistent blood-stained vulvovaginal discharge and intense pruritus over 12 months.​ She is a known smoker and attends colposcopy regularly for HPV-positive smears. There was no significant past medical, surgical, or family history other than three cesarean sections and spina bifida occulta. ​On clinical examination, a sinus tract was seen lateral to the right labia majora accompanied by a 1/1cm firm and palpable swelling above it.​ ​Inflammatory markers were normal, and an MRI pelvis was requested which confirmed a 6-cm long sinus tract running along the right labia major anterosuperior from the skin surface and did not reveal any involvement of deep structures (Figure 1).​

**Figure 2:**
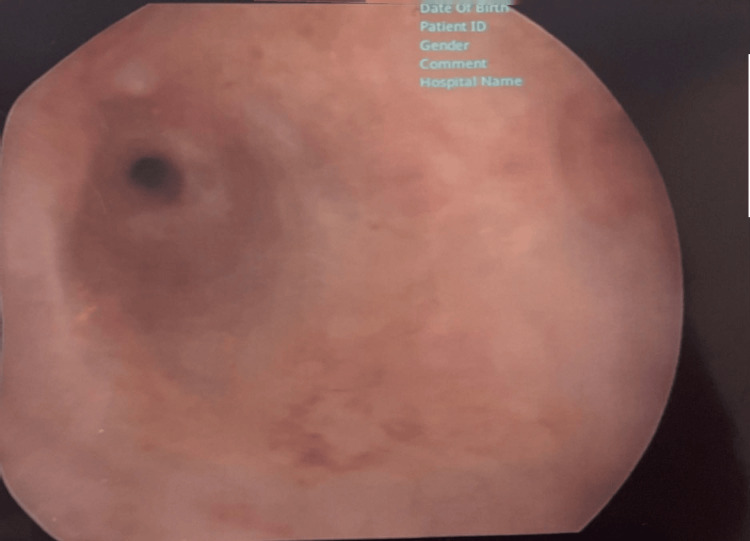
Hysteroscopic view of uterine cavity showing atrophic endometrium

Ultrasound pelvis showed endometrial thickness of 6mm. Hysteroscopy showed an atrophic endometrial cavity excluding endometrial causes of bleeding (Figure 2).

**Figure 3:**
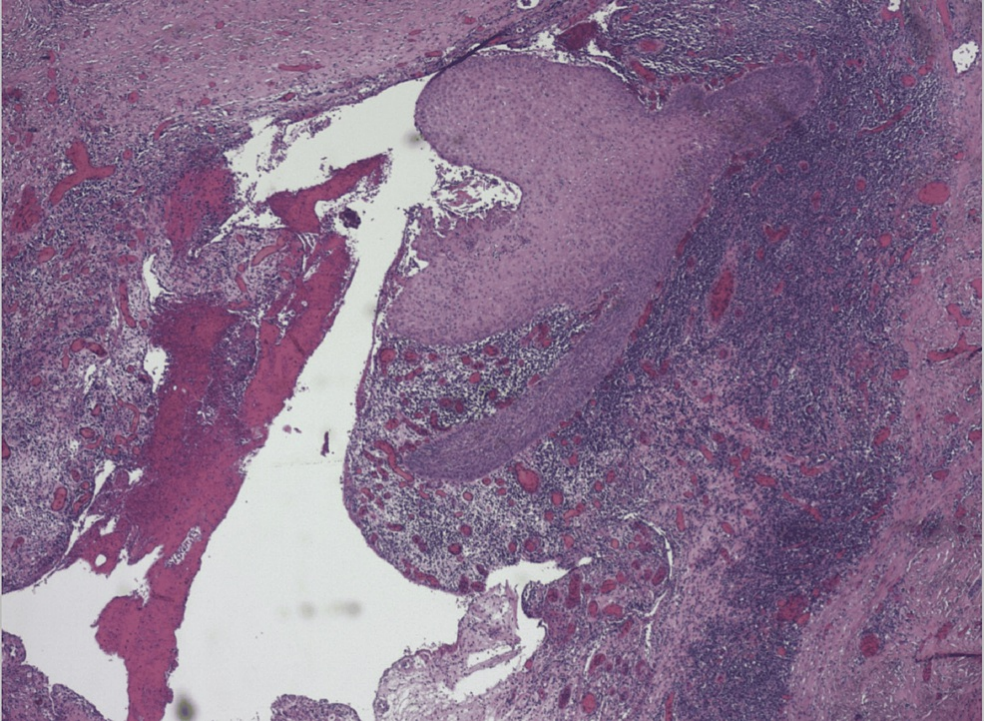
Microphotograph with histological features of sinus tract

Examination under anesthesia revealed an isolated 9 cm sinus tract in the right labiocrural fold, discharging blood-stained yellowish fluid at two points. There was no inguinal lymphadenopathy. An intraoperative general surgical input was taken to out rule extension or involvement of deeper perineal structures. Sinus tract was not extending to the anus, vagina, or perianal area. The tract was completely excised by the team. There were no other tracts or sinuses seen. A clinical diagnosis of hidradenitis suppurativa was made as the discharge resembled sebum. However, histopathology showed skin with a deep dermal sinus tract showing oedematous and heavily inflamed granulation tissue and focal surviving squamous epithelium. There was no evidence of hidradenitis, dysplasia, or malignancy. The presence of a chronic inflamed sinus tract characterized by the absence of hair follicles and apocrine glands out-ruled differentials confirmed the diagnosis in our case. An informed consent has been obtained from the patient (Figure 3).

Outcome and follow-up

Postoperative review at eight weeks showed complete closure of the sinus tract and resolution of symptoms. The patient was followed for one year and did not show any recurrence of signs and symptoms related to this condition.

## Discussion

A vulval sinus could be a blind end track that extends from the skin to an underlying area or cavity [3]. The symptoms of a chronic discharging vulval sinus may include persistent foul-smelling discharge from the vulval opening, swelling and tenderness, dyspareunia, and can be associated with recurrent infections in the vulval region.​ Occasionally it can be asymptomatic. It can develop spontaneously or secondary to tissue damage occurring after vulval trauma, infection, or foreign body, or can be iatrogenic [1]. The pilonidal disease of the female genital ​tract​ causing the formation of the vulval sinus is very rare and disease involving the vulva extremely uncommon with only five cases described in the literature [4-8]. Tubercular labial sinus is a rare but important cause in developing countries. Infection is a common sequel of these factors and can lead to the formation of an abscess. ​Chronic abscesses that become epithelialized eventually become a sinus [1].​ Diagnosing a chronic discharging vulval sinus involves a review of the patient’s symptoms, background medical history, and a detailed physical examination. Additional tests, such as swabs or imaging studies, may be necessary to identify the underlying cause and guide appropriate treatment.

Seeing multiple sinuses at the same or different locations is a common occurrence. ​Therefore, utmost attention should be given to exploring the number, location, and extent of these sinus tracts along with special attention given to any connection to underlying cavities.​ ​A sinus tract should always be assessed using a probe to assess its length, direction, and depth.​ ​Clinically, the external opening is often seen as a small, dimpled area draining pus on manual compression [2].​ Other diagnostic methods to delineate the entire sinus tract are CT scan, MRI, and sonography [1]. ​Treatment of sinus is always focused on treating its cause.​ ​Appropriate antibiotic cover should be given to treat underlying infection.​ ​Any factors causing vulval trauma should be removed.​ Examination under anesthesia may add additional information when direct clinical examination is inconclusive. Complete excision of the sinus tract along with accurate detection of any deep extensions of sinus tracts are paramount for successful treatment. Incomplete excision or missed extensions of the sinus tract can lead to failed treatment, abscess formation, and recurrence of sinus tracts. It can also lead to long-term complications like chronic pain, persistent vulval discharge, perineal scarring, disfigurement, and fistulas. ​Patients who receive multiple conservative management for discharging sinus in the absence of overt sepsis like fever, pain, or other symptoms must be meticulously managed.​ Management of vulval sinus includes local wound opening and drainage, debridement of ischemic tissues, and removal of foreign bodies if present. The definitive treatment of chronic or persistent discharging sinus is a surgical excision of the sinus tract [1]. ​Women should also be advised to take certain self-care measures like maintaining good hygiene practices to reduce the risk of developing a chronic discharging vulval sinus.​ Histopathology of such cases shows acute and chronic inflammation with inflamed granulation tissue. The absence of apocrine glands and hair follicles will help to differentiate it from hidradenitis and pilonidal sinus which are important differentials for vulval sinuses.

## Conclusions

A chronic discharging vulval sinus in women requires careful evaluation and appropriate management to alleviate discomfort and prevent complications including chronic discharge and recurrent abscess formation. Early diagnosis can help women affected by this condition receive effective treatment, leading to improved well-being and a better quality of life. It is crucial to prioritize vulval health and engage in proactive healthcare measures to ensure a positive outlook for women facing such challenges.
